# Sleep Quality Moderates the Impact of Place-Based Social Adversity on Physical Health in Women with Breast Cancer Transitioning from Active Treatment to Survivorship

**DOI:** 10.3390/curroncol32080420

**Published:** 2025-07-26

**Authors:** Crystal L. Park, Katherine E. Gnall, Caroline Salafia, Keith M. Bellizzi

**Affiliations:** 1 Department of Psychological Sciences, University of Connecticut, Storrs, CT 06269, USA; 2 Department of Human Development and Family Sciences, University of Connecticut, Storrs, CT 06269, USA

**Keywords:** breast cancer, place-based social adversity, sleep, cancer survivorship

## Abstract

Social adversity is linked to poorer physical health in breast cancer survivors, highlighting the urgency of addressing health equity. Simultaneously, identifying individual-level factors that mitigate these effects may provide more immediate relief for survivors. This study examined whether four modifiable psychosocial factors—emotion dysregulation, physical activity, sleep disturbance, and social support—moderate the relationship between place-based social adversity and physical health in 255 breast cancer survivors (Mage = 56.03, 74.5% non-Hispanic White) within six months post-treatment. Linear regression analyses with 5000 bootstrapped estimates revealed that sleep disturbance significantly moderated the relationship between place-based social adversity and physical health (B = −0.014, SE = 0.001, bootstrapped 95% CI = −0.027, −0.001). Specifically, greater place-based social adversity was associated with poorer physical health at high levels of sleep disturbance (B = −0.22, *p* = 0.004), but not at low (B = 0.01, *p* = 0.94) or average (B = −0.10, *p* = 0.07) levels. Emotion dysregulation, physical activity, and social support did not moderate this relationship. Findings suggest that improving sleep quality may buffer the negative impact of social adversity on physical health, identifying sleep as a potential target for interventions aimed at reducing disparities among breast cancer survivors.

## Simple Summary

In this study, we explored the role of socially disadvantaged neighborhoods in determining breast cancer survivors’ physical health after treatment. Specifically, we aimed to determine whether certain personal factors—sleep quality, emotional regulation, physical activity, and social support—could help protect survivors from these negative effects. Among the 255 women studied, we found that sleep quality played a key role: women who had trouble sleeping and lived in areas with more social adversity had worse physical health, but this was not the case for women who slept well. The other personal factors studied did not show any protective effect. Our findings suggest that improving sleep may help to reduce health disparities in breast cancer survivors and could guide future research and interventions focused on improving longer-term physical health in survivorship.

## 1. Introduction

Breast cancer is the most commonly diagnosed cancer among women in the United States, accounting for approximately 30% of all new female cancer cases each year [1]. In 2025, an estimated 316,950 new cases of invasive breast cancer and 59,080 cases of ductal carcinoma in situ (DCIS) were projected to be diagnosed in women, with approximately 42,170 women projected to die from the disease [2]. Due to advances in detection and treatment, most women are being diagnosed with breast cancer in the earlier stages of the disease, and as a result, the prognosis for breast cancer has improved greatly over time, with a current five-year survival rate over 90 percent [3]. However, while breast cancer incidence has remained relatively stable in recent years, health disparities persist as demonstrated by the unequal burden of cancer incidence, prevalence, and outcomes among socially disadvantaged groups [1,4]. Therefore, it is essential to investigate modifiable factors that may alleviate the health consequences of social adversity faced by women diagnosed with breast cancer to enhance survivorship outcomes. In this study, we focused specifically on global physical health, which encompasses limitations and impairments related to physical functioning (e.g., ability to perform physical activities such as walking or climbing stairs), role limitations due to physical health problems (e.g., difficulty with work or daily activities), and bodily pain.

Although more women are living longer after a breast cancer diagnosis, the effects of breast cancer and its treatment (e.g., surgery and radiation) can cause physical and physiological side effects, such as fatigue and pain [5,6]. However, the transition from active treatment to cancer survivorship presents an opportunity to intervene and promote behavioral and psychosocial changes that can improve physical health and well-being. Early survivorship is an especially unique transition period in which patients are more open to making lifestyle changes, perhaps as a way to focus on reducing risk of recurrence, taking back control, or managing risk of side effects (e.g., [7]). As such, examining modifiable factors that may mitigate social disadvantages during the early survivorship period is particularly critical for informing targeted intervention efforts. The aim of the present study was to examine whether four modifiable factors (i.e., emotion dysregulation, physical activity, sleep disturbance, and social support) modify (i.e., buffer or exacerbate) the relationship between social adversity and physical health among breast cancer survivors who recently completed their primary cancer treatment.

### 1.1. Social Adversity and Health Consequences of Breast Cancer

The social context in which an individual lives profoundly influences their health and well-being [8,9]. A growing body of literature links various forms of social adversity—defined as chronic or acute exposure to adverse social conditions that threaten physical or psychological functioning—to poorer health outcomes [8,9]. Examples of social adversity include poverty, discrimination, limited access to healthcare, food insecurity, unsafe neighborhoods, and lack of social support. In the context of breast cancer specifically, aspects of social adversity—such as race, income, insurance status, and social network characteristics—have been consistently associated with worse physical health outcomes. The social context in which an individual lives influences their health and well-being [8,9]. A growing literature links aspects of social adversity as reflected in race, income, insurance status, and social networks to poorer physical health outcomes in the context of breast cancer more specifically. Such disparities in breast cancer include disproportionate incidence, later stage at diagnosis, poorer treatment outcomes, and greater mortality [10,11]. Disparities in breast cancer outcomes stem from a variety of social adversity factors, including socioeconomic status, healthcare access and policy, and deeply embedded structural inequities [11]. Chronic stress exposure and the associated physiological changes are additional well-documented mechanisms through which social disadvantages impact physical health and well-being [12].

In addition to social adversity broadly, one specific factor that is emerging as an important determinant of breast cancer outcomes is place-based adversity, particularly neighborhood-level adversity. Neighborhood adversity refers to socioeconomic disadvantage in a local region based on income, education, employment, and housing quality [13,14]. Studies of women with breast cancer have demonstrated that neighborhood disadvantage is related to later stage at diagnosis [15], more complications after post-mastectomy reconstructive surgery [16], and higher mortality rates [17,18]. Neighborhood adversity has also been associated with health-related quality of life across numerous samples of women with breast cancer, including those undergoing chemotherapy [19], breast cancer survivors post-mastectomy [20], and longer-term (1–5 years post-treatment) Black and Hispanic breast cancer survivors [21].

### 1.2. Need to Both Address Health Equity and Identify Individual-Level Moderators

Addressing social adversity is essential for improving public health and reducing health disparities both broadly and within the domain of cancer survivorship. At the societal and policy level, structural inequities such as poverty, discrimination, and limited access to quality healthcare must be addressed through systemic reforms, including policies that promote economic stability, education, and healthcare access [8].

While longer-term efforts toward social justice and health equity are essential, it is also important to simultaneously identify modifiable behavioral and psychosocial factors that can help mitigate the immediate health consequences of social adversity on breast cancer survivors’ physical health. Studies have shown that a variety of behavioral and psychosocial factors predict better physical health and quality of life in breast cancer survivors (for reviews, see [22,23]). Once identified, these psychosocial resources can then serve as treatment targets for interventions—such as cognitive-behavioral strategies, stress management techniques, and patient-centered communication—that can be integrated into healthcare settings [24]. In this way, healthcare professionals can provide immediate, evidence-based support to vulnerable populations of breast cancer survivors while pursuing broader structural changes [25]. This dual approach ensures that while systemic inequities are being addressed, cancer survivors currently experiencing social adversity receive meaningful, accessible interventions that promote well-being and resilience.

### 1.3. Modifiable Psychosocial and Behavioral Factors as Buffers

Based on the current literature regarding predictors of health and well-being in breast cancer survivors (e.g., [23,26]) and the psychosocial model of stress-buffering, which posits that psychosocial and behavioral factors may actually protect vulnerable individuals by mitigating the negative effects of socioeconomic and environmental stressors on physical health and well-being such as social adversity [27], we focused on four modifiable factors: psychosocial factors (emotion regulation and social support) and behavioral factors (physical activity and sleep) that have been related to better health and well-being in breast cancer survivors. We hypothesized that these resources would not only predict better physical health in breast cancer survivors but may also serve as protective factors against the adverse effects of social adversity on physical health for breast cancer survivors. For instance, individuals facing financial hardship or discrimination may experience heightened distress and inflammation, exacerbating health disparities in cancer survivorship [28,29,30]. However, engagement in regular physical activity, maintaining healthy sleep patterns, and leveraging emotional and social resources could attenuate these negative effects by promoting resilience and physiological regulation [31,32]. While each of our four selected psychosocial and behavioral factors are robustly linked to health and well-being in breast cancer survivors, little research has tested them as moderators of the social adversity-health link in this population.

Emotion regulation abilities refer to skills in altering one’s emotional reaction to a stressful event in order to maintain a preferred emotional state [33]. Emotion regulation involves not just modulating emotional arousal but also awareness, understanding, and acceptance of emotions and the ability to act in desired ways regardless of emotional state. Prospective studies in breast cancer patients and survivors have shown that successful emotion regulation predicts improved physical well-being, whereas difficulties in emotion regulation (i.e., emotion dysregulation) relate to disease progression, decreases in physical and psychological function, all-cause mortality, and poor treatment outcomes [34,35,36]. To date, very little research has examined emotion regulation as modifying the impact of social adversity on physical health in the context of any type of cancer, including specifically breast cancer.

It is well-documented that social support plays an important role in health outcomes and disease progression after breast cancer diagnosis and treatment [37,38]. Both direct and indirect relationships between social support and physical health (e.g., pain) in breast cancer survivors are well-established (e.g., [39,40,41,42]). Social support as a buffer in the context of cancer is an intriguing but rarely examined possibility. An earlier analysis of the full sample (including breast, prostate, and colorectal cancer survivors) from which the present study is drawn found that social support moderated the impact of social adversity on comorbidities [43]. However, whether social support moderates the effects of social adversity on physical health in breast cancer survivors who had recently completed primary cancer treatment remains untested.

Physical activity is a well-known facilitator of a host of mental and physical aspects of health and well-being among breast cancer patients and survivors [44] including cognitive functioning during chemotherapy [45], quality of life [46], physical fitness [47], depression and anxiety [48], and mortality (e.g., [49]). However, whether physical activity buffers the associations of social adversity on physical health in breast cancer survivors, or more generally in the context of cancer, is not yet established.

Sleep quality is also a well-established predictor of physical health in breast cancer patients and survivors, including lower fatigue [50] and inflammatory markers [51] in women undergoing chemotherapy, cognitive functioning after chemotherapy [52], higher levels of health-related quality of life [53], and lower mortality [54]. Similar to the abovementioned behavioral and psychosocial factors, however, research has not yet examined whether sleep quality moderates the impact of social adversity on physical health for breast cancer survivors (or, indeed, survivors of any type of cancer).

### 1.4. Study Aim

The aim of this study was to test the moderating roles of emotion dysregulation, social support, physical activity, and sleep disturbance on the relationship between place-based social adversity and physical health among recent breast cancer survivors. We hypothesized that place-based social adversity would predict poorer physical health and that each psychosocial and behavioral factor would buffer these adverse effects.

## 2. Materials and Methods

### 2.1. Participants and Procedures

The current study is a secondary analysis focusing on breast cancer survivors who participated in the YU-CAN study. The YU-CAN study is a longitudinal, observational investigation that tracked 569 adults diagnosed with cancer as they progressed from active treatment into early survivorship [55]. Eligible participants were between the ages of 18 and 80 at the time of diagnosis and had recently been diagnosed with stage I–III breast, prostate, or colorectal cancer, with no prior cancer history. Recruitment was conducted through the Yale Cancer Center’s Rapid Case Ascertainment program. Additional eligibility criteria included being within six months of diagnosis and having the ability to read and speak English.

Participants completed online self-report questionnaires at five timepoints: the initial baseline assessment (within six months of diagnosis) and at subsequent 3-month intervals over the course of one year. To compensate participants for their time, a $50 gift card was provided for each completed survey, with an additional $50 bonus for completing all five assessments. For further details on recruitment and study procedures, see [55]. For the present secondary analysis, a subsample of breast cancer survivors who had completed their primary cancer treatment within the past six months were assessed (*N* = 255). Given that participants completed primary treatment at different timepoints throughout the study, data were then lined up such that each participant’s data for the present secondary analysis corresponded with their first survey completed immediately following their documented primary cancer treatment end date. To standardize the timeframe, participants with greater than six months between the end of treatment and their next subsequent survey were excluded. Additionally, participants who did not have any data for the primary outcomes (i.e., physical health) at the timepoint immediately following the end of cancer treatment (i.e., no available data) or who did not have a defined primary treatment end date were also excluded.

### 2.2. Measures

At baseline, participants reported sociodemographic characteristics, including age, gender, race, ethnicity, and education. Clinical variables, including disease stage at diagnosis, diagnosis date, and treatment information, were extracted from patient medical records.

Place-based social adversity was measured with the Social Deprivation Index (SDI), a composite indicator of area-level social determinants of health. The SDI incorporates seven demographic indicators: the percentage of individuals living in poverty, with less than a high school education, in single-parent households, in rental housing, in overcrowded housing, without access to a vehicle, and non-employed adults under age 65 [56]. Developed to explore links between social disadvantage and health outcomes, the SDI provides a summary measure of neighborhood-level deprivation. Participants’ zip codes, obtained from medical records, were used to assign SDI scores. Values range from 0 to 100, with higher scores indicating greater levels of social deprivation.

Physical health was assessed with the six physical function items of the SF-12. A sample item is, “During the past 4 weeks, how much of the time have you accomplished less than you would have liked to as a result of your physical health? Would you say…” As Likert scales varied slightly for each item, items were recoded to be on a scale from 0–100, with higher scores indicating greater physical health. Responses to the six physical health questions were then averaged to calculate a mean score (possible range 0–100), with higher scores indicating better health [57].

Emotion dysregulation was measured using the 18-item Difficulties in Emotion Regulation Scale—Short Form (DERS-SF; [58]). Participants rated how often each statement applied to them on a 5-point scale ranging from 1 (almost never) to 5 (almost always), with higher scores reflecting greater difficulties in emotion regulation. A sample item is, “I have difficulty making sense out of my feelings.” Internal consistency for the DERS-SF in the current sample was high (Cronbach’s α = 0.90).

Perceived social support was assessed using the 19-item Medical Outcomes Study Social Support Survey (MOS-SS; [59]). This measure captures four dimensions of social support: emotional/informational support, tangible support, affectionate support, and positive social interaction. Responding to the stem “How often is each of the following kinds of support available to you if you need it?”, a sample item is “Someone you can count on to listen to you when you need to talk.” Responses were provided on a 5-point scale from 1 (none of the time) to 5 (all of the time), with higher scores indicating greater perceived social support. Cronbach’s alpha in the present sample was excellent (α = 0.97).

Physical activity was evaluated with the Godin Leisure-Time Exercise Questionnaire [60]. Participants reported the frequency with which they engaged in mild, moderate, and strenuous physical activity for at least 15 min during the past week. A sample item is, “During a typical 7-day period, how many times do you engage in strenuous exercise (heart beats rapidly) for more than 15 min?” A total physical activity score was computed by multiplying the number of weekly bouts by 3 (mild), 5 (moderate), and 9 (strenuous) and summing the weighted values to yield an overall leisure-time physical activity score.

Sleep disturbance was assessed with the Patient-Reported Outcomes Measurement Information System (PROMIS) Sleep Disturbance Short Form 8b [61]. A sample item is, “I had trouble sleeping.” Participants rated the frequency of experiencing each item on a scale from 1 (e.g., not at all, never) to 5 (e.g., very much, always). A total sleep disturbance score was calculated by taking an average of the items and multiplying by eight (possible range 8–40), with higher scores indicating greater sleep disturbance. Cronbach’s alpha in the present sample is 0.94.

### 2.3. Statistical Analysis Plan

SPSS (Version 30) was used for all analyses. First, independent sample *t*-tests (continuous variables) and chi-square tests (categorical variables) were conducted comparing participants with breast cancer who were included in the present secondary analysis to those who were excluded. Next, descriptive statistics were examined, and Pearson bivariate correlations were conducted to examine associations between study variables. Next, separate linear regression models were conducted to examine the moderating role of each psychosocial variable on the relation between SDI and physical function, and models were carefully inspected to ensure they met model assumptions. Both SDI and psychosocial moderators were mean-centered prior to performing regression analyses. The PROCESS macro [62] was used to test the moderating role of each psychosocial variable on the relation between SDI and physical health, and to probe conditional effects of SDI for the significant interactions (i.e., simple slopes analysis). Regression models were first run without the interaction term to allow for accurate interpretation of the independent effects of predictors, and then regression models were run again using PROCESS with the interaction term included. All regression models included chemotherapy (yes/no), radiation (yes/no), hormone therapy (yes/no), and age as covariates, and all models included 5000 bootstrapped samples.

## 3. Results

### 3.1. Sample Characteristics and Bivariate Correlations

Sample characteristics are presented in Table 1. The mean age of participants in this sample was 56.09 (SD = 12.30). The majority of participants identified as White (88.2%) and non-Hispanic (82.4%). Just over half of participants were diagnosed with stage I breast cancer (55.3%), and the average number of days since end of primary cancer treatment was 70.94 (SD = 45.11) (i.e., 2.36 months).

Participants did not significantly differ with regard to age, ethnicity, marital status, education, cancer stage, nor social adversity (all *p*s > 0.34); the only significant difference found was for race (χ^2^ (1, *N* = 332) = 12.65, *p* < 0.001), suggesting that participants who identified as White were more likely to be included in the secondary analysis compared to those who did not identify as White.

Bivariate correlations are reported in Table 2. SDI was significantly associated with higher levels of sleep disturbance (*p* = 0.02) and worse physical health (*p* = 0.001), but not with age nor the three other psychosocial moderators (all *p*s > 0.05). Better physical health was associated with fewer emotion regulation difficulties and sleep disturbance as well as greater social support and physical activity (all *p*s < 0.01).

### 3.2. Moderation Analyses

Emotion Dysregulation Model: Prior to adding the interaction term, both emotion dysregulation (bootstrapped 95% CI = −19.32, −7.59) and SDI (bootstrapped 95% CI = −0.27, −0.05) demonstrated significant independent associations with physical health. The interaction between emotion dysregulation and SDI on physical health was not significant (bootstrapped 95% CI = −0.26, 0.08).

Social Support Model: Prior to adding the interaction term, higher social support was independently associated with better physical health (bootstrapped 95% CI = 0.27, 0.58), while SDI was not associated with physical health when including social support in the model (bootstrapped 95% CI = −0.23, 0.01). The interaction between social support and SDI on physical health was not significant (bootstrapped 95% CI = −0.004, 0.01).

Physical Activity Model: Prior to adding the interaction term, both physical activity (bootstrapped 95% CI = 0.20, 0.49) and SDI (bootstrapped 95% CI = −0.27, −0.04) demonstrated significant independent associations with physical health. The interaction between physical activity and SDI on physical health was not significant (bootstrapped 95% CI = −0.001, 0.003). Of note, one potential residual outlier was identified. A sensitivity analysis was conducted to examine models with vs. without that participant, and results remained consistent. As such, we retained the model with all participants included.

Sleep Disturbance Model: Prior to adding the interaction term, both sleep disturbance (bootstrapped 95% CI = −1.36, −0.51) and SDI (bootstrapped 95% CI = −0.24, −0.01) demonstrated significant independent associations with physical health. Additionally, the interaction between SDI and sleep disturbance was significant (B = −0.01, SE = 0.01, bootstrapped 95% CI = −0.03, −0.0002). A simple slopes analysis revealed that at high (1SD above mean) sleep disturbance, higher SDI was associated with lower physical health (B = −0.21, *p* = 0.01). SDI was not associated with physical health at low (B = 0.01, *p* = 0.96) nor average (B = −0.10, *p* = 0.08) sleep disturbance.

**Figure 1:**
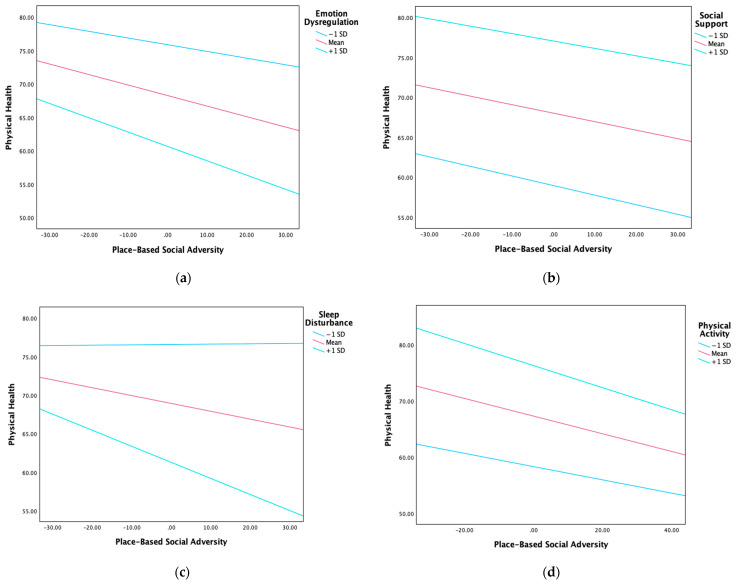
Psychosocial factors as moderators of the relationship between place-based social adversity and physical health. Note. (**a**) emotion dysregulation as moderator; (**b**) social support as moderator; (**c**) sleep disturbance as moderator; (**d**) physical activity as moderator. Interaction effect is significant only for graph c (sleep disturbance model). All other interaction effects are not statistically significant.

Results of moderation analyses and graphs of interaction effects are reported in Table 3 and Figure 1, respectively.

## 4. Discussion

In the present study, we tested whether four modifiable psychosocial and behavioral factors—emotion dysregulation, social support, physical activity, and sleep disturbance—modify (buffer or exacerbate) the relationship between place-level social adversity and physical health among breast cancer survivors recently completing primary treatment. In line with our hypothesis, place-based social deprivation was significantly associated with poorer physical health, even after accounting for age and treatment-related variables. This finding aligns with prior research demonstrating that social adversity more broadly is a critical determinant of disparities in cancer outcomes, including poorer treatment response and survivorship quality [11,18]. However, of the four hypothesized moderators, only sleep disturbance emerged as a significant moderator of the association between place-based social adversity and physical health.

This study is among the first to demonstrate that sleep disturbance may exacerbate the adverse impact of place-based social deprivation on physical health among breast cancer survivors. Specifically, the relationship between greater place-based social adversity and poorer physical health was significantly stronger among survivors reporting higher levels of sleep disturbance. This finding is consistent with existing evidence that sleep plays a critical role in physical recovery and inflammation regulation, particularly in populations already at risk due to stress or illness [51,54]. These findings extend prior work by showing that sleep disturbance not only predicts poorer physical health in general but also amplifies the negative consequences of social adversity on health during a critical transition point in cancer survivorship. Importantly, this finding suggests that improving sleep during early survivorship may be a particularly effective target for interventions designed to reduce health disparities among breast cancer survivors facing social disadvantages. However, it is also critical to note that sleep disturbance itself is impacted by social adversity factors. For example, neighborhood factors such as noise pollution or excessive heat, as well as lack of air conditioning or soundproofing may affect ability to sleep well [63,64]. As such, targeting sleep disturbance in individuals facing social disadvantage requires awareness and consideration of an individual’s broader social context.

Contrary to our hypotheses, emotion dysregulation, social support, and physical activity did not significantly moderate the relationship between place-based social adversity and physical health in this sample. These null findings have several potential explanations. One possibility is that while these factors are broadly associated with better quality of life in breast cancer survivors [34,48], they may not be potent enough to buffer the specific effects of neighborhood-level adversity, which reflects more structural and systemic disadvantage. Additionally, our measure of physical health captured general daily physical capabilities such as pain and ability to engage in social activities and household chores but may not have been sensitive enough to detect more specific changes associated with psychosocial resources.

It is also possible that the buffering effects of emotion regulation, social support, and physical activity may be more apparent in other phases of survivorship. For example, survivors might diverge as survivorship time lengthens, allowing for the emergence of more individual differences in buffering effects. Buffering effects might also emerge in populations with more diverse sociodemographic characteristics. Our sample was largely White and non-Hispanic, limiting variability in both adversity exposure and psychosocial resources. Moreover, prior research suggests that emotion regulation and social support may have stronger effects on psychological outcomes (e.g., depression and anxiety) than on physical health per se [36,41], highlighting the importance of outcome specificity when testing moderators.

Critically, we want to be clear that (a) individual-level psychosocial and behavioral factors themselves are highly impacted by social adversity, and (b) targeting individual-level factors cannot divert attention away from enacting policies affecting broader societal-level change top-down [65]. Rather, identifying modifiable individual-level factors offers an additional more immediate target to support vulnerable populations while moving towards broader structural changes.

Our findings have both clinical and public health relevance and support a dual-level approach to health equity in cancer survivorship: addressing systemic sources of inequality while concurrently providing immediate, targeted behavioral interventions to support at-risk individuals [8,25]. Specifically, these findings reinforce the need to address neighborhood-level social disadvantage as a key determinant of post-treatment physical health in breast cancer survivors. Additionally, they suggest that interventions targeting sleep disturbance may be particularly impactful for improving physical health and reducing disparities in this vulnerable population. Sleep-focused interventions such as cognitive-behavioral therapy for insomnia (CBT-I) are scalable and have demonstrated efficacy in cancer populations [66] making them a promising treatment for survivorship care programs, particularly for patients residing in high-deprivation neighborhoods. As mentioned above, sleep disturbance is also impacted by social adversity, requiring interventions responsive to an individual’s social context. For instance, digital delivery of CBT-I may reduce key barriers to accessing evidence-based treatment for sleep disturbance [67].

Additionally, the present study captured breast cancer survivors early in survivorship (i.e., within 6 months of completing active cancer treatment). Given that the early survivorship period is a key time for behavior change [7], interventions targeting sleep specifically during the early survivorship period may capitalize on patients’ openness to change to deliver downstream improvements in functioning and quality of life. Future research should examine whether sleep-focused interventions differentially benefit cancer survivors experiencing higher levels of social adversity and whether improvements in sleep translate into sustained gains in physical health.

Several limitations of our study should be noted. First, the cross-sectional nature of this analysis precludes drawing conclusions about causality. Longitudinal research is needed to confirm whether sleep disturbance amplifies the effect of social adversity on physical health over time. Second, the sample was predominantly White and well-educated, limiting generalizability to more diverse populations who may experience greater levels of social adversity. Examining these questions in more socioeconomically and racially diverse samples is essential for improving generalizability and informing equity-focused intervention strategies. Third, place-based social disadvantage was derived from zip-code level data, which may not fully capture more localized or subjective aspects of adversity (e.g., safety and community cohesion) nor an individual’s specific circumstances. Furthermore, we conducted four separate models, raising the possibility of Type I error. Finally, although our psychosocial moderators were selected based on strong theoretical and empirical rationale, other unmeasured variables—such as self-efficacy, access to healthcare, or financial toxicity—may play important but unaddressed roles in our findings.

## 5. Conclusions

Overall, this study adds to the growing evidence that place-based social adversity contributes to poorer health outcomes among breast cancer survivors and identifies sleep disturbance as a potential amplifier of these effects. While broader structural changes are needed to address health inequities, these findings highlight the potential promise of sleep as a modifiable target for mitigating the health consequences of social adversity during the critical period of early survivorship. Tailored, accessible behavioral interventions—such as making materials culturally-specific (e.g., [68])—may help reduce disparities and promote resilience among breast cancer survivors facing disproportionate burdens of social disadvantage.

## Figures and Tables

**Table 1:** Sample characteristics (*N* = 255).

Variable	*M* (*SD*) or *N* (%)	Range
Age	56.09 (12.30)	24–80
Race ^a^		
White	225 (88.2%)	-
Black or African American	15 (5.9%)	-
American Indian or Alaska Native	7 (2.7%)	-
Asian	5 (2.0%)	-
Not reported or unknown	4 (1.6%)	-
Ethnicity		
Hispanic/Latinx	19 (7.5%)	-
Not Hispanic/Latinx	210 (82.4%)	-
Not reported or unknown	26 (10.2%)	-
Highest level of education		
No formal education	1 (0.4%)	-
Grade school	2 (0.8%)	-
High school graduate	21 (8.2%)	-
Some college or associate degree	66 (25.9%)	-
Bachelor’s degree	68 (26.7%)	-
Graduate or professional degree	5 (2.0%)	-
Master’s degree	72 (28.2%)	-
Doctoral degree or professional degree	15 (5.9%)	-
Not reported	5 (2.0%)	-
Breast cancer stage		
I	141 (55.3%)	-
II	90 (35.3%)	-
III	19 (7.5%)	-
Missing	5 (2.0%)	-
SDI	29.59 (29.60)	1.00–98.00
Physical health	68.54 (28.03)	0.00–100.00
Emotion dysregulation	1.87 (0.59)	1.00–4.50
Social support	78.57 (21.52)	11.84–100.00
Sleep disturbance	23.45 (8.19)	8.00–40.00
Physical activity ^b^	29.06 (26.20)	0.00–123.00

Note. Age missing for *n* = 1.^a^ Participants could choose all that apply, so race total is greater than 100%; ^b^ physical activity is reported as total leisure time score in the past week based on the Godin Leisure Time Physical Activity Questionnaire.

**Table 2:** Bivariate correlations.

	1	2	3	4	5	6
1. Age	-					
2. Social adversity	.01	-				
3. Emotion dysregulation	−.25 **	−.01	-			
4. Social support	.06	−.10	−.31**	-		
5. Physical activity	−.19 **	−.07	.07	.13	-	
6. Sleep disturbance	−.22 **	.14 *	.34 **	−.24 **	.03	-
7. Physical health	.07	−.21 **	−.28 **	.33 **	.30 **	−.30 **

* *p* < 0.05, ** *p* < 0.01.

**Table 3:** Psychosocial moderators of the relationship between place-based social adversity and physical health.

	Emotion Dysregulation Model			Social Support Model			Sleep Disturbance Model			Physical Activity Model		
	B	SE	Bootstrapped 95% CI	B	SE	Bootstrapped 95% CI	B	SE	Bootstrapped 95% CI	B	SE	Bootstrapped 95% CI
Age	−0.15	0.16	−0.46, 0.17	0.01	0.16	−0.30, 0.32	−0.10	0.15	−0.40, 0.20	0.05	0.16	−0.27, 0.37
Chemotherapy	**−7.51**	**3.49**	**−14.30, −0.55**	−6.62	3.43	−13.45, 0.22	−5.71	3.43	−12.44, 0.97	−6.34	3.70	−13.33, 1.12
Radiation	**11.13**	**4.07**	**3.10, 19.32**	**14.63**	**4.31**	**6.19, 23.01**	**10.62**	**4.14**	**2.22, 18.68**	**12.31**	**4.28**	**3.83, 20.68**
Hormone therapy	0.82	4.20	−7.53, 9.15	−1.64	4.16	−9.50, 6.41	3.69	4.18	−4.45, 11.89	2.29	4.18	−5.69, 10.36
Social adversity	**−0.16**	**0.06**	**−0.27, −0.05**	−0.11	0.06	−0.23, 0.01	**−0.12**	**0.06**	**−0.24, −0.01**	**−0.16**	**0.06**	**−0.27, −0.04**
Psychosocial factor	**−13.25**	**3.04**	**−19.32, −7.59**	**0.42**	**0.08**	**0.27, 0.58**	**−0.94**	**0.22**	**−1.36, −0.51**	**0.34**	**0.07**	**0.20, 0.49**
Social adversity × psychosocial factor	−0.10	0.09	−0.26, 0.08	0.001	0.003	−0.004, 0.01	**−0.01**	**0.01**	**−0.03, −0.0002**	−0.002	0.002	−0.001, 0.003

Note. To allow for accurate interpretation of the independent coefficient estimates of each variable, multiple regression models were first run without the interaction term (Step 1), and then again including the interaction term using the PROCESS Macro (Step 2). Demographic variables (age, chemotherapy, radiation, hormone therapy) included as covariates in all models. All models include 5000 bootstrapped samples. Statistically significant results are reported in bold font.

## Data Availability

The raw data supporting the conclusions of this article will be made available by the authors on reasonable request.

## References

[1] American Cancer Society Cancer Facts & Figures 2025. https://www.cancer.org/research/cancer-facts-statistics/all-cancer-facts-figures/2025-cancer-facts-figures.html.

[2] Siegel R.L., Kratzer T.B., Giaquinto A.N., Sung H., Jemal A. (2025). Cancer statistics, 2025. CA Cancer J. Clin..

[3] Soldato D., Arecco L., Agostinetto E., Franzoi M.A., Mariamidze E., Begijanashvili S., Brunetti N., Spinaci S., Solinas C., Vaz-Luis I. (2023). The Future of Breast Cancer Research in the Survivorship Field. Oncol. Ther..

[4] Thompson B., Hohl S.D., Molina Y., Paskett E.D., Fisher J.L., Baltic R.D., Washington C.M. (2018). Breast cancer disparities among women in underserved communities in the USA. Curr. Breast Cancer Rep..

[5] Parker P.A., Youssef A., Walker S., Basen-Engquist K., Cohen L., Gritz E.R., Wei Q.X., Robb G.L. (2007). Short-term and long-term psychosocial adjustment and quality of life in women undergoing different surgical procedures for breast cancer. Ann. Surg. Oncol..

[6] Shapiro C.L., Recht A. (2001). Side effects of adjuvant treatment of breast cancer. N. Engl. J. Med..

[7] Frazelle M.L., Friend P.J. (2016). Optimizing the teachable moment for health promotion for cancer survivors and their families. J. Adv. Pract. Oncol..

[8] Braveman P., Egerter S., Williams D.R. (2011). The social determinants of health: Coming of age. Annu. Rev. Public Health.

[9] Garg A., Jack B., Zuckerman B. (2013). Addressing the social determinants of health within the patient-centered medical home: Lessons from pediatrics. JAMA.

[10] Veličković K., Borrebaeck C.A.K., Bendahl P.-O., Hegardt C., Johnsson P., Richter C., Rydén L., Hallberg I.R. (2022). One-year recovery from breast cancer: Importance of tumor and treatment-related factors, resilience, and sociodemographic factors for health-related quality of life. Front. Oncol..

[11] Williams A.D., Moo T.-A. (2023). The impact of socioeconomic status and social determinants of health on disparities in breast cancer incidence, treatment, and outcomes. Curr. Breast Cancer Rep..

[12] McClendon J., Chang K., JBoudreaux M., Oltmanns T.F., Bogdan R. (2021). Black-White racial health disparities in inflammation and physical health: Cumulative stress, social isolation, and health behaviors. Psychoneuroendocrinology.

[13] Chamberlain A.M., Finney Rutten L.J., Wilson P.M., Fan C., Boyd C.M., Jacobson D.J., Rocca W.A., Sauver J.L.S. (2020). Neighborhood socioeconomic disadvantage is associated with multimorbidity in a geographically-defined community. BMC Public Health.

[14] Kind A.J.H., Buckingham W.R. (2018). Making neighborhood-disadvantage metrics accessible—The neighborhood atlas. N. Engl. J. Med..

[15] Babatunde O.A., Zahnd W.E., Eberth J.M., Lawson A.B., Adams S.A., Boakye E.A., Jefferson M.S., Allen C.G., Pearce J.L., Li H. (2021). Association between neighborhood social deprivation and stage at diagnosis among breast cancer patients in South Carolina. Int. J. Environ. Res. Public Health.

[16] Wang C., Frost J., Tang M., Shah R., Kim E., Shamamian P.E., Montalmant K.E., Oleru O., Seyidova N., Henderson P.W. (2024). Neighborhood deprivation is associated with increased postoperative complications after implant-based breast reconstruction. Clin. Breast Cancer.

[17] Barber L.E., Maliniak M.L., Nash R., Moubadder L., Haynes D., Ward K.C., McCullough L.E. (2024). A comparison of three area-level indices of neighborhood deprivation and socioeconomic status and their applicability to breast cancer mortality. J. Urban Health.

[18] Roy A.M., George A., Attwood K., Alaklabi S., Patel A., Omilian A.R., Yao S., Gandhi S. (2023). Effect of Neighborhood Deprivation Index on breast cancer survival in the United States. Breast Cancer Res. Treat..

[19] You K.-L., Sereika S.M., Bender C.M., Hamilton J.B., Mazanec S.R., Brufsky A., Rosenzweig M.Q. (2024). Health-related quality of life over chemotherapy course among individuals with early-stage breast cancer: The association of social determinants of health and neighborhood socioeconomic disadvantage. Support. Care Cancer.

[20] Hassan A.M., Nguyen H.T., Corkum J.P., Liu J., Kapur S.K., Chu C.K., Tamirisa N., Offodile A.C. (2023). Area deprivation index is associated with variation in quality of life and psychosocial well-being following breast cancer surgery. Ann. Surg. Oncol..

[21] Wu C., Ashing K.T., Jones V.C., Barcelo L. (2018). The association of neighborhood context with health outcomes among ethnic minority breast cancer survivors. J. Behav. Med..

[22] Culbertson M.G., Bennett K., Kelly C.M., Sharp L., Cahir C. (2020). The psychosocial determinants of quality of life in breast cancer survivors: A scoping review. BMC Cancer.

[23] Pezzolato M., Spada G.E., Fragale E., Cutica I., Masiero M., Marzorati C., Pravettoni G. (2023). Predictive models of psychological distress, quality of life, and adherence to medication in breast cancer patients: Aa scoping review. Patient Prefer. Adherence.

[24] Wells K.J., Drizin J.H., Ustjanauskas A.E., Vázquez-Otero C., Pan-Weisz T.M., Ung D., Carrizosa C., Laronga C., Roetzheim R.G., Johnson K. (2022). The psychosocial needs of underserved breast cancer survivors and perspectives of their clinicians and support providers. Support. Care Cancer.

[25] Gallo L.C., Matthews K.A. (2003). Understanding the association between socioeconomic status and physical health: Do negative emotions play a role?. Psychol. Bull..

[26] Syrowatka A., Motulsky A., Kurteva S., Hanley J.A., Dixon W.G., Meguerditchian A.N., Tamblyn R. (2017). Predictors of distress in female breast cancer survivors: A systematic review. Breast Cancer Res. Treat..

[27] Umberson D., Montez J.K. (2010). Social relationships and health: A flashpoint for health policy. J. Health Soc. Behav..

[28] Saban K.L., Mathews H.L., Bryant F.B., Tell D., Joyce C., DeVon H.A., Janusek L.W. (2018). Perceived discrimination is associated with the inflammatory response to acute laboratory stress in women at risk for cardiovascular disease. Brain Behav. Immun..

[29] Sturgeon J.A., Arewasikporn A., Okun M.A., Davis M.C., Ong A.D., Zautra A.J. (2016). The psychosocial context of financial stress: Implications for inflammation and psychological health. Psychosom. Med..

[30] Zheng Z., Han X., Zhao J., Banegas M.P., Tucker-Seeley R., Rai A., Fedewa S.A., Song W., Jemal A., Yabroff K.R. (2020). Financial hardship, healthcare utilization, and health among U.S. cancer survivors. Am. J. Prev. Med..

[31] Mahindru A., Patil P., Agrawal V. (2023). Role of physical activity on mental health and well-being: A review. Cureus.

[32] Martín-Rodríguez A., Gostian-Ropotin L.A., Beltrán-Velasco A.I., Belando-Pedreño N., Simón J.A., López-Mora C., Navarro-Jiménez E., Tornero-Aguilera J.F., Clemente-Suárez V.J. (2024). Sporting mind: The interplay of physical activity and psychological health. Sports.

[33] Thompson R.A. (1994). Emotion regulation: A theme in search of definition. Monogr. Soc. Res. Child. Dev..

[34] Brandão T., Tavares R., Schulz M.S., Matos P.M. (2016). Measuring emotion regulation and emotional expression in breast cancer patients: A systematic review. Clin. Psychol. Rev..

[35] Baziliansky S., Cohen M. (2021). Emotion regulation and psychological distress in cancer survivors: A systematic review and meta-analysis. Stress Health.

[36] Conley C.C., Bishop B.T., Andersen B.L. (2016). Emotions and emotion regulation in breast cancer survivorship. Healthcare.

[37] Finck C., Barradas S., Zenger M., Hinz A. (2018). Quality of life in breast cancer patients: Associations with optimism and social support. Int. J. Clin. Health Psychol..

[38] Cheng H., Sit J.W.H., Chan C.W.H., So W.K.W., Choi K.C., Cheng K.K.F. (2013). Social support and quality of life among Chinese breast cancer survivors: Findings from a mixed methods study. Eur. J. Oncol. Nurs..

[39] Chou A.F., Stewart S.L., Wild R.C., Bloom J.R. (2012). Social support and survival in young women with breast carcinoma. Psychooncology.

[40] Hughes S., Jaremka L.M., Alfano C.M., Glaser R., Povoski S.P., Lipari A.M., Farrar W.B., Yee L.D., Carson W.E., Malarkey W.B. (2014). Social support predicts inflammation, pain, and depressive symptoms: Longitudinal relationships among breast cancer survivors. Psychoneuroendocrinology.

[41] Zhang H., Zhao Q., Cao P., Ren G. (2017). Resilience and quality of life: Exploring the mediator role of social support in patients with breast cancer. Med. Sci. Monit..

[42] Thompson T., Pérez M., Kreuter M., Margenthaler J., Colditz G., Jeffe D.B. (2017). Perceived social support in African American breast cancer patients: Predictors and effects. Soc. Sci. Med..

[43] Bellizzi K.M., Fritzson E., Ligus K., Park C.L. (2024). Social support buffers the effect of social deprivation on comorbidity burden in adults with cancer. Ann. Behav. Med..

[44] de Boer M.C., Wörner E.A., Verlaan D., van Leeuwen P.A.M. (2017). The mechanisms and effects of physical activity on breast cancer. Clin. Breast Cancer.

[45] Koevoets E.W., Schagen S.B., de Ruiter M.B., Geerlings M.I., Witlox L., van der Wall E., Stuiver M.M., Sonke G.S., Velthuis M.J., Jobsen J.J. (2022). Effect of physical exercise on cognitive function after chemotherapy in patients with breast cancer: A randomized controlled trial (PAM study). Breast Cancer Res..

[46] Bucciarelli V., Bianco F., Di Blasio A., Morano T., Tuosto D., Mucedola F., Di Santo S., Cimini A., Napolitano G., Bucci I. (2023). Cardiometabolic profile, physical activity, and quality of life in breast cancer survivors after different physical exercise protocols: A 34-month follow-up study. J. Clin. Med..

[47] Hooshmand Moghadam B., Golestani F., Bagheri R., Cheraghloo N., Eskandari M., Wong A., Nordvall M., Suzuki K., Pournemati P. (2021). The effects of high-intensity interval training vs. Moderate-intensity continuous training on inflammatory markers, body composition, and physical fitness in overweight/obese survivors of breast cancer: A randomized controlled clinical trial. Cancers.

[48] Rogers L.Q., Courneya K.S., Anton P.M., Verhulst S., Vicari S.K., Robbs R.S., McAuley E. (2017). Effects of a multicomponent physical activity behavior change intervention on fatigue, anxiety, and depressive symptomatology in breast cancer survivors: Randomized trial. Psychooncology.

[49] Spei M.-E., Samoli E., Bravi F., La Vecchia C., Bamia C., Benetou V. (2019). Physical activity in breast cancer survivors: A systematic review and meta-analysis on overall and breast cancer survival. Breast.

[50] Ratcliff C.G., Lam C.Y., Arun B., Valero V., Cohen L. (2014). Ecological momentary assessment of sleep, symptoms, and mood during chemotherapy for breast cancer. Psychooncology.

[51] Liu L., Mills P.J., Rissling M., Fiorentino L., Natarajan L., Dimsdale J.E., Sadler G.R., Parker B.A., Ancoli-Israel S. (2012). Fatigue and sleep quality are associated with changes in inflammatory markers in breast cancer patients undergoing chemotherapy. Brain Behav. Immun..

[52] Henneghan A.M., Carter P., Stuifbergan A., Parmelee B., Kesler S. (2018). Relationships between self-reported sleep quality components and cognitive functioning in breast cancer survivors up to 10 years following chemotherapy. Psychooncology.

[53] Lourenço A., Dantas A.A.G., de Souza J.C., Araujo C.M., Araujo D.N., Lima I.N.D.F., Dantas D.d.S. (2020). Sleep quality is associated with Disability and Quality of life in breast cancer survivors: A cross-sectional pilot study. Eur. J. Cancer Care.

[54] Trudel-Fitzgerald C., Zhou E.S., Poole E.M., Zhang X., Michels K.B., Eliassen A.H., Chen W.Y., Holmes M.D., Tworoger S.S., Schernhammer E.S. (2017). Sleep and survival among women with breast cancer: 30 years of follow-up within the Nurses’ Health Study. Br. J. Cancer.

[55] Bellizzi K.M., Park C.L., Lee J.W., Harel O., Sanft T., Fritzson E., Salafia C., Ligus K., Gnall K., Magin Z.E. (2025). Physical health and function trajectories in adults with cancer: Psychosocial predictors of class membership. J. Cancer Surviv..

[56] Robert Graham Center (2018). Social Deprivation Index (SDI). Robert Graham Center. https://www.graham-center.org/maps-data-tools/social-deprivation-index.html.

[57] Hagell P., Westergren A., Årestedt K. (2017). Beware of the origin of numbers: Standard scoring of the SF-12 and SF-36 summary measures distorts measurement and score interpretations. Res. Nurs. Health.

[58] Kaufman E.A., Xia M., Fosco G., Yaptangco M., Skidmore C.R., Crowell S.E. (2016). The difficulties in emotion regulation scale short form (DERS-SF): Validation and replication in adolescent and adult samples. J. Psychopathol. Behav. Assess..

[59] Sherbourne C.D., Stewart A.L. (1991). The MOS social support survey. Soc. Sci. Med..

[60] Godin G. (2011). The Godin-Shephard leisure-time physical activity questionnaire. Health Fitness J. Can..

[61] Yu L., Buysse D.J., Germain A., Moul D.E., Stover A., Dodds N.E., Johnston K.L., Pilkonis P.A. (2011). Development of short forms from the PROMIS^TM^ sleep disturbance and Sleep-Related Impairment item banks. Behav. Sleep Med..

[62] Hayes A.F. (2017). Introduction to Mediation, Moderation, and Conditional Process Analysis: A Regression-Based Approach.

[63] Patel N.P., Grandner M.A., Xie D., Branas C.C., Gooneratne N. (2010). “Sleep disparity” in the population: Poor sleep quality is strongly associated with poverty and ethnicity. BMC Public Health.

[64] Sosso F.A.E., Holmes S.D., Weinstein A.A. (2021). Influence of socioeconomic status on objective sleep measurement: A systematic review and meta-analysis of actigraphy studies. Sleep Health.

[65] Seabrook J.A., Avison W.R. (2012). Socioeconomic status and cumulative disadvantage processes across the life course: Implications for health outcomes. Can. Rev. Sociol..

[66] Johnson J.A., Rash J.A., Campbell T.S., Savard J., Gehrman P.R., Perlis M., Carlson L.E., Garland S.N. (2016). A systematic review and meta-analysis of randomized controlled trials of cognitive behavior therapy for insomnia (CBT-I) in cancer survivors. Sleep Med. Rev..

[67] Cheng P., Luik A.I., Fellman-Couture C., Peterson E., Joseph C.L.M., Tallent G., Tran K.M., Ahmedani B.K., Roehrs T., Roth T. (2019). Efficacy of digital CBT for insomnia to reduce depression across demographic groups: A randomized trial. Psychol. Med..

[68] Zhou E.S., Ritterband L.M., Bethea T.N., Robles Y.P., Heeren T.C., Rosenberg L. (2022). Effect of culturally tailored, internet-delivered cognitive behavioral therapy for insomnia in Black women: A randomized clinical trial. JAMA Psychiatry.

